# Hepatic progesterone receptor membrane component 1 attenuates ethanol-induced liver injury by reducing acetaldehyde production and oxidative stress

**DOI:** 10.1152/ajpgi.00206.2022

**Published:** 2023-04-18

**Authors:** Seong-Lae Jo, In-Jeoung Baek, Je-Won Ko, Hyo-Jung Kwun, Hyun-Jin Shin, Eui-Ju Hong

**Affiliations:** ^1^College of Veterinary Medicine, Chungnam National University, Daejeon, Korea; ^2^Department of Convergence Medicine, University of Ulsan College of Medicine, Asan Medical Center, Seoul, Republic of Korea

**Keywords:** alcohol-associated liver disease, ER stress, PGRMC1

## Abstract

Alcohol-associated liver disease (ALD) is caused by excessive abuse of alcohol. One of the most representative causes of ALD is the action of acetaldehyde. Acetaldehyde is a toxic material produced when alcohol is metabolized through some enzymes, and it causes endoplasmic reticulum (ER) stress, mitochondrial dysfunction, and tissue injury. In this study, we assessed the relationship between Progesterone receptor membrane component 1 (PGRMC1) and ALD because PGRMC1 is expressed in the ER and mitochondria in the liver. Using the chronic and binge alcohol feeding models, we assessed acetaldehyde level, liver damage, alcohol-degrading enzymes, and ER stress. Compared with wild-type (WT) mice ethanol-fed *Pgrmc1* knockout (KO) mice had higher levels of alanine aminotransferase (ALT) and alcohol-degrading enzymes, and *Pgrmc1* KO mice had high serum acetaldehyde and ER stress levels compared with WT mice with control and ethanol feeding. Loss of *Pgrmc1* increased acetaldehyde production through increased expression of alcohol dehydrogenase and catalase, which led to increased ER stress and suggested that cell death was promoted. In conclusion, it has been proposed that the loss of PGRMC1 could promote ALD and cause liver damage in alcohol-abusing humans.

**NEW & NOTEWORTHY** Loss of *Pgrmc1* increased acetaldehyde production, and excess acetaldehyde consequently increased ER stress, which activates apoptosis. Since low expression of *PGRMC1* is vulnerable to alcoholic liver damage, the loss of *PGRMC1* expression may increase susceptibility to ALD.

## Graphical abstract

**Figure:**
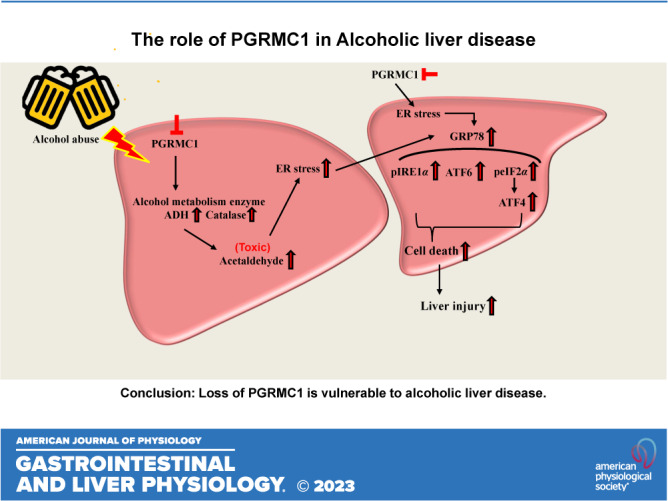

## INTRODUCTION

Approximately 2 billion people worldwide consume alcohol; of these, ∼75 million are diagnosed with alcohol abuse and alcohol-associated liver disease (ALD), including severe liver disease and liver damage. The World Health Organization reports that globally the trends in the per capita consumption of alcohol by active drinkers increased between 2000 and 2016, and alcohol abuse was reported to cause 3 million deaths worldwide in 2016. ALD mortality increased between 2006 and 2020 (1), and alcohol consumption increased substantially in the United States during the COVID-19 pandemic (2). Overall, the increase in alcohol consumption may have led to alcohol abuse, to which the increase in ALD-associated mortality could be attributed.

Similar to nonalcoholic fatty liver disease (NAFLD), ALD includes alcoholic fatty liver, alcoholic steatohepatitis, alcoholic hepatitis, alcoholic cirrhosis, and hepatocellular cancer (3). The early stage of alcoholic liver disease is characterized by alcoholic fatty liver or steatosis, which involves fat accumulation in the liver cells. Alcoholic hepatitis occurs in patients with high alcohol intake and critical alcoholic fatty liver or fibrosis (4). If alcohol abstinence, nutritional support, treatment of infection, and drug therapy fail in hepatitis, it can lead to alcoholic cirrhosis (5).

The alcoholic cirrhosis stage is irreversible and leads to various complications (6). However, even though a positive correlation has been reported between cumulative alcohol intake and the degree of ALD (7), the associated environmental risk factors and genetic factors need to be considered (8, 9).

Although ALD and NAFLD are similar with respect to pathological findings and pathogenesis, ALD is mediated by acetaldehyde (ACA) during alcohol metabolism, whereas NAFLD is mediated via steatosis, which involves an increase in the plasma triglyceride (TG) levels via de novo adipogenesis in response to a high daily caloric intake (3, 10).

Alcohol dehydrogenase (ADH), cytochrome *P*-450 2E1 (CYP2E1), and catalase are alcohol-degrading enzymes. Alcohol is converted to ACA by ADH, CYP2E1, and catalase, which is then converted to acetate by aldehyde dehydrogenase (11). ALD is induced by ACA, reactive oxygen species (ROS), and cytokines and in response to nutritional intake disorders (12). One of the most representative causes of ALD is the action of ACA. ACA is a toxic molecule produced when alcohol is metabolized by enzymes. It causes endoplasmic reticulum (ER) stress, mitochondrial dysfunction, and tissue injury (13). ACA damages the mitochondria, ER, and cytoplasm of hepatocytes. We hypothesized that progesterone receptor membrane component 1 (*Pgrmc1*) knockout (KO) mice fed a liquid ethanol (EtOH) diet would exhibit more severe liver damage in response to EtOH metabolites and oxidative stress because they produce ACA at higher levels and exhibit ER stress, both of which induce cytotoxicity in various tissues (14–16).

Progesterone receptor membrane component 1 (PGRMC1) is known to be in various tissues and highly expressed in the human liver and kidneys (17). As the primary regulator for hepatic de novo lipogenesis, sterol regulatory element-binding protein 1 (Srebp-1) complex is transmitted from the PGRMC1, implying that PGRMC1 is closely involved with SREBP-1 function (18). Based on our previous study (19), *Pgrmc1* KO mice are predisposed to steatohepatitis and nonalcoholic fatty liver disease (NAFLD). Moreover, *Pgrmc1* KO could predispose to hepatocellular carcinoma via low epidermal growth factor receptor expression and inflammatory cytokines (20). We also reported that progesterone-mediated PGRMC1 expression induced cyclic adenosine monophosphate-mediated phosphoenolpyruvate carboxykinase induction and glucose production in the liver (21). Recently, it was reported that PGRMC1 binds and stabilizes various cytochrome *P*-450 families in the liver (22), affecting drug, hormone, and lipid metabolism (23). However, to the best of our knowledge, the role of Pgrmc1 in ALD has not been addressed in any study. The PGRMC1 protein is localized to the plasma membrane and endomembrane organelles, including the ER, nuclear membrane, endosomes, and Golgi bodies (24). In hepatic cells, PGRMC1 may regulate the expression of alcohol-degrading enzymes and stabilize cytochrome *P*-450. Interestingly, we showed that *Pgrmc1* KO mice exhibited increased ER stress in the liver in both the basal and alcohol-fed states. ER stress increases in response to alcohol stimulation (13); acute or chronic alcohol exposure leads to the generation of reactive oxygen species, thus inducing ER stress and ALD development (25). In light of this evidence, to assess the role of Pgrmc1 in ALD we used the National Institute on Alcohol Abuse and Alcoholism (NIAAA) model (for chronic and binge alcohol feeding) for inducing ALD in whole body *Pgrmc1* KO mice.

## MATERIALS AND METHODS

### Animals and Mouse Modeling

As reported previously (19), *Pgrmc1* whole body KO mice and wild-type (WT) mice were used in this study and were housed in a pathogen-free facility at Chungnam National University under a standard 12:12-h light-dark cycle and fed standard chow with water provided ad libitum. All mouse experiments were approved and performed in accordance with the Chungnam Facility Animal Care Committee (202006 CNU-105). We used male mice to minimize variability in the effects of female sex hormones because the female hormones influence PGRMC1 expression. We mimicked the chronic-binge ethanol (EtOH) feeding model (the NIAAA model). In EtOH groups, 8- to 10-wk-old male WT and *Pgrmc1* KO mice were fed a liquid control diet (DYET, Bethlehem, PA) on the first day of the acclimatization period, followed by a progressive increase in EtOH concentration from 1% to 4% (vol/vol) from *day 2* to *day 5*. Then we fed a liquid diet containing 5% EtOH for 10 days, and control groups were fed a control diet for 10 days. At *day 11*, the mice in the EtOH groups were gavaged with a single dose of EtOH (7 g/kg body wt, 30% EtOH), whereas the mice in the control groups were gavaged with maltodextrin containing the same number of calories as the EtOH gavage. Mice were euthanized at 9 h after gavage (26). The number of mice used for the experiment was 7 for each group.

### Primary Hepatocyte Culture

Primary cell culture was performed as described previously (21). Primary hepatocytes were isolated from mice by collagenase digestion and Percoll gradient method. First, mice were anesthetized, and the peritoneal cavity was opened. Livers were perfused with Ca^2+^- and Mg^2+^-free HBSS containing EDTA (1 mM) (LB203-56; Welgene) and then digested with a collagenase solution containing Liberase (Liberase TM Research Grade; Sigma). Digested livers were removed and rinsed twice with HBSS and then gently teased with forceps until they were in solution. The cell suspensions were filtered through a sterile 40-μm nylon cell strainer to remove undigested and connective tissue. The cells were centrifuged for 5 min at 1,000 rpm and resuspended with medium. The pellet suspensions were centrifuged with 35% Percoll for 15 min at 2,000 rpm with the brake option off. After centrifugation the healthy hepatocytes were pelleted, as damaged hepatocytes or nonparenchymal cells could not penetrate into 35% Percoll solution. The pellets were washed twice with DMEM supplemented with 5% FBS and then seeded into six-well tissue culture plates. After 24 h, nonadherent cells were removed by aspiration.

### Western Blotting

Both protein samples of the liver were extracted with a protein lysis buffer called T-PER reagent (78510; Thermo Fisher Scientific, Waltham, MA) and quantified by Bradford assay with PRO-MEASURE solution (no. 21011; Intron, Kirkland, WA). In addition, the serum sample was diluted with PBS and the experiment was conducted. Depending on the protein size, the samples were run with SDS-PAGE electrophoresis on 10% or 12% polyacrylamide gels and transferred to the membrane. The membranes were blocked for 1 h with 3% BSA100 (9048-46-8; LPS Solution, Daejeon, Korea), diluted TBS-Tween (TBS-T) buffer (04870517TBST4021; LPS Solution). Primary antibodies were incubated overnight at 4°C. After this step, the membranes were washed with TBS-T, and the secondary antibodies were incubated in an identical way. Results were detected with ECL solution (XLS025-0000; Cyanagen, Bologna, Italy) and Chemi Doc (Fusion Solo; VilberLourmat, Collégien, France). The primary antibodies were diluted at 1:2,500 in 5% (wt/vol) BSA, and the secondary antibodies were diluted at 1:2,500 in 5% (wt/vol) skim milk. The primary and secondary antibody information is in Supplemental Table S1.

### Total RNA Extraction and Real-Time Quantitative PCR

Total RNA was extracted with TRIzol reagent (15596-026; Life Technologies) in both mouse livers. Reverse transcription was performed with 1.5 µg of total RNA and a reverse transcriptase kit (SG-cDNAS100; Smartgene) according to the manufacturer’s protocol. Quantitative PCR (real-time PCR) was executed with Excel Taq Q-PCR Master Mix (SG-SYBR-500; Smartgene) and Stratagene Mx3000P (Agilent Technologies). The primers used in real-time PCR were manufactured by Bionics Inc. (Seoul, Korea). RPLP0 was used as a control in in vivo samples. All experiments were run at least in triplicate, and mRNA values were calculated based on the cycle threshold and monitored for an amplification curve. The primers used for real-time PCR are shown in Supplemental Table S2.

### Serum Triglyceride, Alanine Aminotransferase, Free Fatty Acid, and Acetaldehyde and Endotoxin Level

Serum triglyceride (TG) and alanine aminotransferase (ALT) levels were measured with FUJI DRI-CHEM SLIDE (TG-1650, ALT-3250) by DRI-CHEM4000 (Fuji Film), Serum free fatty acid (FFA) was measured with a commercial kit (BM-FFA100; Biomax). ACA level was measured with a colorimetric kit (EACT-100; BioAssay Systems), and endotoxin (lipopolysaccharide) level was measured with a colorimetric kit (A39552; Thermo Fisher Scientific, Inc.)

### Liver Triglyceride Level

Lipid extraction from the liver was done with the Folch method. Liver tissue (0.04 g) was homogenized with beads and 0.9% NaCl solution. After being mixed with chloroform and methanol (1:2), samples were allowed to stand for 30 min at room temperature. Additionally mixed with chloroform and distilled water, the lower phase was separated after being centrifuged at 3,000 rpm, for 20 min. Steps after homogenization were repeated three times. Samples were filtrated with filter paper, and the flow-through was heated in the water bath. By this step, the chloroform phase was evaporated. After being completely dried in a dry oven for 3 h, it was dissolved in PBS. Samples were stored at room temperature. TG level was analyzed with TG measurement solution (AM157S-K; Asan-Set) in a 550-nm spectrophotometer.

### Histological and Oil Red O Analyses

For hematoxylin and eosin (H&E) staining, paraffin-embedded tissues were cut to 4 μm and attached to silane-coated slides. With a serial hydration step in xylene, EtOH, and distilled water, hematoxylin and eosin stains were applied. The region of interest was observed with a light microscope. For Oil Red O, frozen tissues were cut to 8 μm after being embedded with OCT compound and attached to silane-coated slides. After being dried for 10 min at room temperature (RT), slides were fixed with formalin for 20 min and washed with running tap water for 10 min. Slides were proceeded to rinse step with 60% isopropanol and stained with Oil Red O working solution for 15 min. After being washed with 60% isopropanol, slides were stained with hematoxylin for 30 s and rinsed with distilled water. The region of interest was observed with a light microscope after being mounted in an aqueous mountant.

### Immunohistochemistry

For immunohistochemistry staining, paraffin-embedded tissues were cut to 4 μm and attached to silane-coated slides, with a serial hydration step in xylene, EtOH, and distilled water and then a progressed antigen retrieval step. The tissue slides were blocked for 1 h with 3% BSA (9048-46-8; LPS Solution), diluted TBS-T buffer (04870517TBST4021; LPS Solution). Primary antibodies were incubated overnight at 4°C. After this step, the membranes were washed with TBS-T, and the Alexa-Fluor secondary antibodies (no. A21471, no. A21207; Thermo Fisher Scientific, Inc.) were incubated identically. After being washed, the slides were mounted in ProLong Gold antifade reagent (P36935; Thermo Fisher Scientific, Inc.) and examined with a DMi8 microscope (Leica Microsystems).

### Statistical Analysis

Data are reported as means ± SD. Differences between means were obtained by Student’s *t* test and one-way ANOVA followed by a Dunnett post analysis performed with Graph Pad software (GraphPad, Inc., San Diego, CA).

## RESULTS

### *Pgrmc1* KO Mice Are Prone to Ethanol-Induced Damage

**Figure 1.**
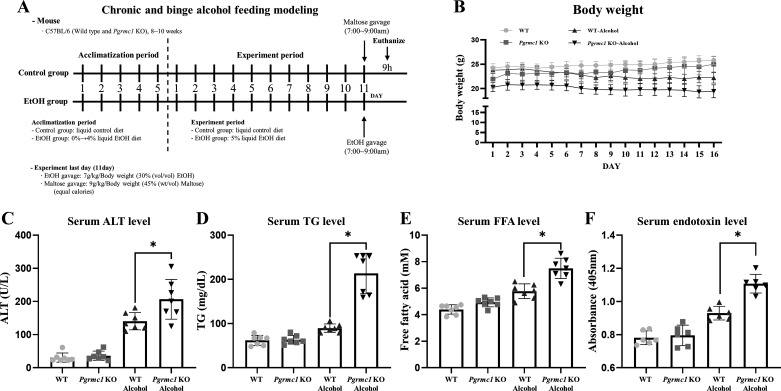
Liver of Progesterone receptor membrane component 1 (*Pgrmc1*) knockout (KO) mouse is vulnerable to ethanol-induced damage. *A*: schematic diagram shows the schedule of an animal experiment. Eight- to ten-week-old male mice had an acclimation period for 5 days. For 11 days, the ethanol (EtOH) group was supplied the liquid EtOH diet and the control group was supplied the liquid control diet. Before being euthanized, the mice in the EtOH groups were gavaged with a single dose of EtOH (7 g/kg body wt, 30% EtOH), whereas the mice in the control groups were gavaged with maltodextrin containing the same number of calories as the EtOH gavage (*n* = 7). *B*: mouse body weight monitoring data. WT, wild type. *C*: serum alanine aminotransferase (ALT) level measured after euthanasia. Serum ALT level was increased in *Pgrmc1* KO mice fed the liquid EtOH diet. *D*: serum triglyceride (TG) level measured after euthanasia. Serum TG level was increased in *Pgrmc1* KO mice fed the liquid EtOH diet. *E*: serum free fatty acid (FFA) level measured after euthanasia. Serum FFA level was increased in *Pgrmc1* KO mice fed the liquid EtOH diet. *F*: serum endotoxin level measured after euthanasia. Serum endotoxin level was increased in *Pgrmc1* KO mice fed the liquid EtOH diet. Values are means ± SD (*n* = 7 in control-fed WT and KO groups, *n* = 7 in EtOH-fed WT and KO groups). *P < 0.05 compared with groups indicated. All experiments were repeated at least 3 times.

To investigate the role of Pgrmc1 in ALD, 8- to 10-wk-old male WT and *Pgrmc1* KO mice were subjected to chronic-binge feeding. The groups of mice had equal calorie intake from the liquid control diet or the liquid EtOH diet (Fig. 1*A*). The body weights of the mice were monitored before euthanasia and analyzed to confirm basic metabolic changes (Fig. 1, *B–F*). Whereas the body weights of the WT and *Pgrmc1* KO mice did not change with intake of the liquid control diet, mice from both groups showed a reduction in body weight with intake of the liquid EtOH diet (*P* < 0.05, 86% in WT mice and 78% in *Pgrmc1* KO mice) compared with mice fed the liquid control diet (Fig. 1*B*). The serum ALT, FFA, TG, and endotoxin levels did not differ between WT mice fed the liquid control diet and *Pgrmc1* KO mice fed the control diet. However, the serum ALT (1.47-fold), TG (2.38-fold), FFA (1.30-fold), and endotoxin (1.19-fold) levels increased in *Pgrmc1* KO mice fed the liquid EtOH diet compared with those in WT mice fed the liquid EtOH diet (*P* < 0.05, Fig. 1, *C*–*F*). We observed that *Pgrmc1* KO mice were susceptible to liver damage after EtOH intake.

### Loss of *Pgrmc1* Expression Induces a Phenotype Characterized by Induction of ADH and Catalase following CYP2E1 Reduction

**Figure 2.**
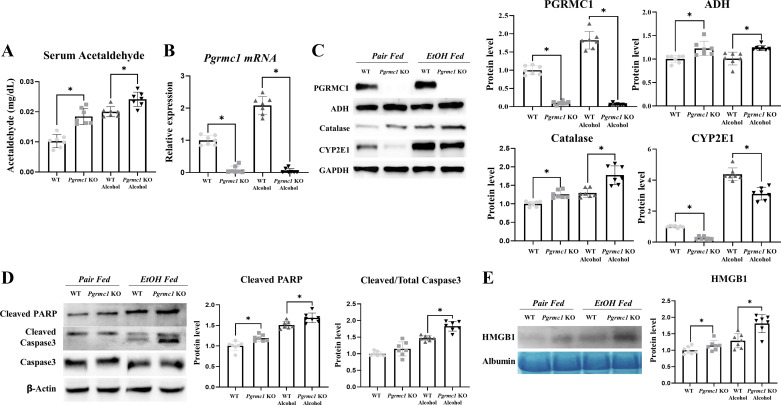
Loss of Progesterone receptor membrane component 1 (*Pgrmc1*) shows phenotype for alcohol-metabolizing enzyme expression. *A*: serum acetaldehyde level was increased in *Pgrmc1* knockout (KO) mice. WT, wild type. *B*: *Pgrmc1* mRNA levels were measured by quantitative RT-PCR of the liver tissue of male mice. RPLP0 was used as an internal control. [*n* = 7 in control-fed WT and KO groups, *n* = 7 in ethanol (EtOH)-fed WT and KO groups]. *C*: Western blot analysis and quantification of PGRMC1 and alcohol-degrading enzymes were performed in the liver tissue of male mice (*n* = 7 in control-fed WT and KO groups, *n* = 7 in EtOH-fed WT and KO groups). GAPDH was used as an internal control. ADH, alcohol dehydrogenase. *D*: Western blot analysis and quantification of apoptosis-related markers were performed in the liver tissue of male mice. β-Actin was used as an internal control. PARP, poly ADP-ribose polymerase. *E*: Western blot analysis and quantification of HMGB1 were performed in the serum of male mice. Albumin was used as an internal control. Values are means ± SD. *P < 0.05 compared with groups indicated. All experiments were repeated at least 3 times.

We showed that serum ALT, FFA, TG, and endotoxin levels were increased in *Pgrmc1* KO mice fed the liquid EtOH diet compared with those in WT mice fed the liquid EtOH diet. Alcohol intake is known to increase serum ALT, TG, FFA, and endotoxin levels (27–30) by increasing levels of ACA. For this reason, we measured serum ACA levels. The serum ACA levels in *Pgrmc1* KO mice increased (*P* < 0.05, 1.21-fold) compared with those in WT mice fed the liquid control diet (Fig. 2*A*). This increase (*P* < 0.05, 1.21-fold) also occurred in *Pgrmc1* KO mice fed the liquid EtOH diet compared with WT mice fed the liquid EtOH diet (Fig. 2*A*). We investigated whether the expression of alcohol-metabolizing enzymes in the liver increased or decreased with the loss of *Pgrmc1* expression. The *Pgrmc1* mRNA levels were higher in WT mice fed the liquid EtOH diet (*P* < 0.05, 2.09-fold) than in WT mice fed the liquid control diet (Fig. 2*B*). In addition, the PGRMC1 levels in the protein extracts from the liver tissues of WT mice fed the liquid EtOH diet were higher (*P* < 0.05, 1.82-fold) than those of WT mice fed the liquid control diet (Fig. 2*C*). We confirmed that *Pgrmc1* was completely depleted in the *Pgrmc1* KO mice (Fig. 2, *B* and *C*). The expression of ADH and catalase proteins was significantly higher (*P* < 0.05, 1.22-fold and 1.24-fold, respectively) in *Pgrmc1* KO mice than in WT mice fed the liquid control diet. Furthermore, the ADH and catalase protein levels were significantly higher (1.22-fold and 1.24-fold, *P* < 0.05) in *Pgrmc1* KO mice than in WT mice fed the liquid EtOH diet. However, the CYP2E1 protein levels were significantly lower in *Pgrmc1* KO mice fed the liquid control diet (26%) and liquid EtOH diet (71%) than in WT mice (*P* < 0.05; Fig. 2*C*). To confirm that the hepatocytes of *Pgrmc1* KO mice have phenotypes of alcohol-metabolizing enzymes, we introduced a primary hepatocyte culture system and observed higher levels of ADH and catalase proteins (1.35-fold and 2.18-fold, respectively, *P* < 0.05) in *Pgrmc1* KO mouse hepatocytes than in WT mouse hepatocytes. In addition, the CYP2E1 protein level was lower in *Pgrmc1* KO hepatocytes (45%, *P* < 0.05) than in WT hepatocytes (Supplemental Fig. S2).

Thus, we showed that the loss of Pgrmc1 expression increased or decreased the expression of alcohol-metabolizing enzymes in the mouse liver. We showed that *Pgrmc1* KO mice are vulnerable to alcohol exposure. Heavy alcohol intake over long periods could cause severe liver damage through apoptosis and necrosis (31). We thus measured apoptosis- and necrosis-related markers in the mice. The levels of the apoptosis-related marker cleaved poly ADP-ribose polymerase (PARP) were significantly higher in *Pgrmc1* KO mice fed the liquid control diet (1.20-fold) and the liquid EtOH diet (1.11-fold) than in WT mice (*P* < 0.05; Fig. 2*D*). The cleaved caspase-3-to-caspase-3 ratio did not differ between the WT and *Pgrmc1* KO mice fed the liquid control diet. However, the cleaved caspase-3-to-caspase-3 ratio was higher in *Pgrmc1* KO mice (*P* < 0.05, 1.24-fold) than in WT mice fed the liquid EtOH diet (Fig. 2*D*).

Compared with WT mice, the necrosis-related marker HMGB1 was significantly upregulated in *Pgrmc1* KO mice fed the liquid control diet (1.14-fold) and the liquid EtOH diet (1.41-fold) (*P* < 0.05; Fig. 2*E*). These results indicate that the loss of *Pgrmc1* expression caused more extensive liver damage in mice fed the liquid EtOH diet (24).

### *Pgrmc1* KO Mice Increase Expression of ER Stress after EtOH Intake

**Figure 3.**
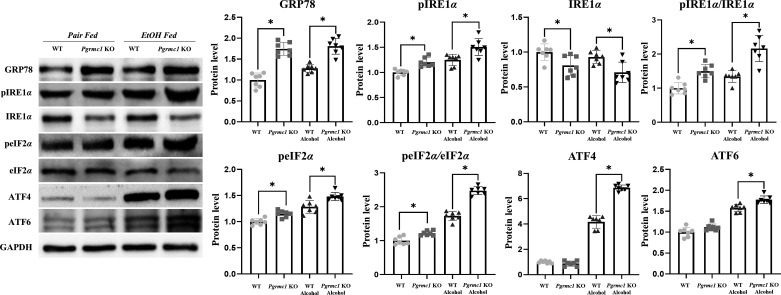
Loss of Progesterone receptor membrane component 1 (*Pgrmc1*) increases endoplasmic reticulum (ER) stress. Western blot analysis and quantification of ER stress-related marker were performed in the liver tissue of male mice. GAPDH was used for an internal control. Values are means ± SD [*n* = 7 in control-fed wild-type (WT) and knockout (KO) groups, *n* = 7 in ethanol (EtOH)-fed WT and KO groups]. *P < 0.05 compared with the groups indicated. All experiments were repeated at least 3 times.

A greater intake of alcohol causes cell death by inducing various forms of cellular stress, including oxidative stress, hypoxia, and ER stress (32). Since PGRMC1 is known to be localized to the ER, we focused on one of the multiple forms of cellular stress: ER stress. To this end, we measured the expression of ER stress-related markers. The levels of GRP78 protein were significantly higher in *Pgrmc1* KO mice fed the liquid control diet (1.75-fold) and the liquid EtOH diet (1.75-fold) than in WT mice (*P* < 0.05; Fig. 3). The phospho- (p)IRE1α protein levels were higher in *Pgrmc1* KO mice (*P* < 0.05, 1.20-fold) than in WT mice fed the liquid control diet. Moreover, the pIRE1α protein levels were higher in *Pgrmc1* KO mice (*P* < 0.05, 1.20-fold) than in WT mice fed the liquid EtOH diet (Fig. 3). The IRE1α protein levels were significantly lower in Pgrmc1 KO mice fed the liquid control diet (81%) and the liquid EtOH diet (76%) than in WT mice (*P* < 0.05; Fig. 3). In summary, the pIRE1α-to-IRE1α ratio was significantly higher in *Pgrmc1* KO mice fed the liquid control diet (1.50-fold) and the liquid EtOH diet (1.61-fold) than in WT mice (*P* < 0.05; Fig. 3). The ATF4 protein level was significantly higher in *Pgrmc1* KO mice fed the liquid EtOH diet (*P* < 0.05, 1.65-fold) than in WT mice fed the liquid EtOH diet (Fig. 3). The peIF2α protein level was significantly higher in *Pgrmc1* KO mice fed the liquid EtOH diet (*P* < 0.05, 1.15-fold) than in WT mice fed the liquid EtOH diet. Moreover, the peIF2α-to-eIF2α ratio was significantly higher in *Pgrmc1* KO mice fed the liquid EtOH diet (*P* < 0.05, 1.43-fold) than in WT mice (Fig. 3). The ATF6 protein levels were significantly higher in *Pgrmc1* KO mice fed the liquid control diet (1.12-fold) and the liquid EtOH diet (1.19-fold) than in WT mice (*P* < 0.05; Fig. 3).

### *Pgrmc1* KO Mice Are Susceptible to Inflammation

**Figure 4.**
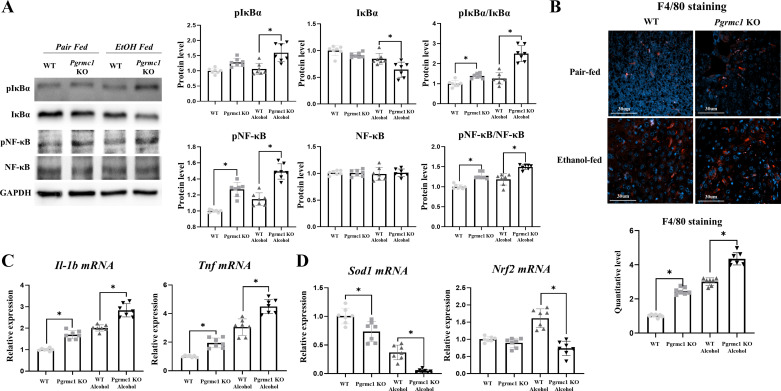
Loss of Progesterone receptor membrane component 1 (*Pgrmc1*) is susceptible to inflammation. *A*: Western blot analysis and quantification of proinflammatory markers were performed in the liver tissue of male mice. GAPDH was used as an internal control. EtOH, ethanol; KO, knockout; WT, wild type. *B*: murine macrophages are characterized in F4/80 staining. Scale bar, 30 µm. Quantification of F4/80 staining was analyzed by ImageJ, setting white holes for positive standard. *C*: *Il-1b* and *Tnf* mRNA levels were measured by quantitative RT-PCR in the liver tissue of male mice. RPLP0 was used as an internal control. *D*: *Sod1* and *Nrf2* mRNA levels were measured by quantitative RT-PCR in the liver tissue of male mice. RPLP0 was used as an internal control. Values are means ± SD (*n* = 7 for each group). *P < 0.05 compared with groups indicated. All experiments were repeated at least 3 times.

Inflammation is known to increase in response to ER stress. To investigate whether inflammation was induced by the loss of *Pgrmc1* expression, we analyzed the expression of proinflammatory markers in the liver tissues. The phospho-IκB protein levels did not differ significantly between WT and *Pgrmc1* KO mice fed a liquid control diet. However, the phospho-IκB protein levels were higher in *Pgrmc1* KO mice (*P* < 0.05, 1.50-fold) than in WT mice fed the liquid EtOH diet (Fig. 4*A*). The IκB protein levels did not differ significantly between WT and *Pgrmc1* KO mice fed the liquid control diet. However, the IκB protein levels were lower in *Pgrmc1* KO mice fed the liquid EtOH diet (*P* < 0.05, 76%) than in WT mice fed the liquid EtOH diet (Fig. 4*A*). The phospho-IκB-to-IκB ratio was higher in *Pgrmc1* KO mice fed the liquid control diet (1.38-fold) and the liquid EtOH diet (1.98-fold) than in WT mice (*P* < 0.05; Fig. 4*A*).

The phospho- (p)NF-κB levels increased in *Pgrmc1* KO mice fed the liquid control diet (1.27-fold) and the liquid EtOH diet (1.30-fold) compared with those in WT mice (*P* < 0.05; Fig. 4*A*). However, the NF-κB protein levels did not differ significantly between WT and *Pgrmc1* KO mice. Overall, the pNF-κB-to-NF-κB ratio was higher in *Pgrmc1* KO mice fed the control diet (1.28-fold) and the liquid EtOH diet (1.26-fold) than in WT mice (*P* < 0.05; Fig. 4*A*). When Kupffer cells were stained with F4/80 antibody, the positive signals were higher in *Pgrmc1* KO mice fed the control diet (2.4-fold) and the liquid EtOH diet (1.6-fold) than in WT mice fed the control diet and the liquid EtOH diet, respectively (*P* < 0.05; Fig. 4*B*). IL-1b and TNF mRNA expression were higher in *Pgrmc1* KO mice fed the liquid control diet (*Il-1b*: 1.70-fold, *Tnf*: 1.94-fold) and the liquid EtOH diet (*Il-1b*: 1.53-fold, *Tnf*: 1.47-fold) than in WT mice (*P* < 0.05; Fig. 4*C*). Reportedly, Nrf2 activation reduces immune cell penetration and decreases the expression of the proinflammatory cytokines TNF-α, IL-1β, and IL-6 by inhibiting the NF-κB signaling pathway (33, 34). We analyzed the expression of antioxidant enzymes because the levels of inflammation-related markers were increased in *Pgrmc1* KO mice. *Sod1* mRNA expression was lower in *Pgrmc1* KO mice fed the liquid control diet (74%) and the liquid EtOH diet (13%) than in WT mice (*P* < 0.05; Fig. 4*D*). The *Nrf2* mRNA expression levels were lower in *Pgrmc1* KO mice (*P* < 0.05, 46%) than in WT mice fed the liquid EtOH diet (Fig. 4*D*).

## DISCUSSION

We observed that the loss of Pgrmc1 expression can increase the risk of ALD and hepatocyte damage under ethanol stimulation compared with that observed in WT mice. When ethanol is metabolized by hepatic alcohol-degrading enzymes, including ADH, CYP2E1, and catalase, the plasma ACA content increases, and ACA induces liver damage (11, 35). ACA is toxic and carcinogenic and binds to proteins, leading to structural and functional changes. In addition to the toxic effects of ACA, cell damage is caused by the direct action of alcohol. ACA- and alcohol-induced structural alterations in the mitochondria lead to functional impairments, including a reduction in ATP formation in the respiratory chain, production of reactive oxygen species, and a decrease in ACA dehydrogenase activity (36). As an indicator of alcohol-induced liver damage, the serum ACA levels were higher in *Pgrmc1* KO mice than in WT mice. Although the ACA levels are primarily affected by environmental conditions (37), the levels of alcohol-degrading enzymes change the ACA production rate based on genetic differences (38). In this study, *Pgrmc1* KO mice showed a relatively low level of CYP2E1 expression and higher ADH and catalase expression compared with WT mice.

It was reported that PGRMC1 binds and stabilizes various cytochrome *P*-450 families in the liver (22), affecting drug, hormone, and lipid metabolism (23). PGRMC1 shares a structural motif with cytochrome *b*5 and can activate cytochrome *P*-450 (39). Although CYP2E1 is one of the primary hepatic alcohol-degrading enzymes, *Pgrmc1* KO was shown with a low level of CYP2E1 expression in the mouse liver tissues. Since it was reported that the ethanol-induced pathologies in *Cyp2e1* KO mice are similar to those observed in WT mice and that CYP2E1 activity is not correlated with oxidative stress in individuals with alcoholism (40), we suggest that a reduction in CYP2E1 expression in *Pgrmc1* KO mice may not be a major contributing factor in ALD.

When the CYP2E1 level of *Pgrmc1* KO mice was relatively lower than that of WT mice, ADH protein of the *Pgrmc1* KO condition was complementarily increased in our models. According to a previous report (41), the high-activity ADH variants would increase the rate of acetaldehyde production. The overexpression of ADH enhances hepatotoxicity caused by ethanol and may cause the accumulation of plasma ACA (38). Catalase can oxidize ethanol to ACA in an H_2_O_2_-dependent manner (35, 42). The ability of catalases to metabolize ethanol may increase in response to oxidative stress with an increase in hepatocellular H_2_O_2_ generation (35). Therefore, the induction of hepatic ADH and catalase expression in *Pgrmc1* KO mice could be associated with the high hepatotoxicity of ACA in the blood (43). These results show that the loss of PGRMC1 expression may exert a fatal effect by causing liver damage via an imbalance in the expression of alcohol-degrading enzymes.

Alcohol intake increases the levels of ACA, which in turn increases serum ALT, TG, FFA, and endotoxin levels (27–30). Serum analysis revealed that the ALT, TG, FFA, and endotoxin levels were higher in *Pgrmc1* KO mice than in WT mice fed the liquid EtOH diet. The levels of serum indicators (ALT, TG, FFA, and endotoxin) increase when ALD is triggered (44, 45). Excessive alcohol consumption leads to disorders in the metabolic function of the liver; the initial stage is generally characterized by fat accumulation in liver cells, leading to fatty liver or steatosis (4). Based on the increase in SREBP-cleavage-activating protein (SCAP)/SREBP-1 expression under low PGRMC1/INSIG-1 expression (18), we previously reported that SREBP-1 increases along with *Pgrmc1* KO in liver tissues in NAFLD induced by a high-fat diet (19). In light of this evidence, we expected that *Pgrmc1* KO mice would be more susceptible to alcoholic fatty liver development induced by intake of a liquid ethanol diet than WT mice. With intake of the liquid EtOH diet, alcoholic fatty liver was induced in both WT and *Pgrmc1* KO mice, as observed by H&E and Oil Red O staining of the tissues. However, the quantitative staining levels did not differ between WT mice fed the liquid ethanol diet and *Pgrmc1* KO mice fed the liquid ethanol diet (Supplemental Fig. S1, A and B). When we measured lipogenesis mediators, including SREBP1 and acetyl-CoA carboxylase, the expression of these markers did not differ significantly between WT and Pgrmc1 mice fed the liquid EtOH diet (Supplemental Fig. S1C). The hepatic TG levels increased in *Pgrmc1* KO mice (*P* < 0.05, 1.21-fold) compared with those in WT mice fed the liquid control diet (Supplemental Fig. S1D). The lack of an observed change in fat accumulation may well be because the model uses a relatively short exposure to EtOH.

Nevertheless, the release of ALT into the bloodstream is the most obvious evidence of liver damage (46) and indicates its association with the increased permeability of the hepatocellular membrane, liver tissue damage, and inflammation (47). On this basis, we speculated that cell death would be high in *Pgrmc1* KO mice. We measured apoptosis- and necrosis-related markers to assess whether the loss of Pgrmc1 expression increased cell death. The expression of the markers increased in *Pgrmc1* KO mice compared with that in WT mice fed the liquid EtOH diet. These findings suggest that the loss of *Pgrmc1* expression increases susceptibility to cell death. Alcohol-induced liver cell death may be induced by various agents, such as ACA and oxidative stress, some of which influence ER injury (13).

We focused on ER stress and apoptosis because *PGRMC1* is present in the ER and mitochondria of liver cells (24). Consistent with these phenotypic characteristics, *Pgrmc1* KO mice showed increased expression of GRP78, which is a master regulator of ER homeostasis. GRP78 contributes to all three major unfolded protein responses (the IREα, ATF6, and PERK signaling pathways) (48), which leads to ER stress (49) and eventually to cell death (50). Therefore, we suggest that the loss of *Pgrmc1* expression increases GRP78 levels, which activates the ER stress detectors and increases the susceptibility of the cells to apoptosis caused by ER stress. Whereas IRE1α induces the biosynthesis of the ER membrane and cell homeostasis, activated IRE1α [phospho-(p)IRE1α] activates a proapoptotic pathway and apoptosis signaling through interaction with various molecules (51, 52). Our results showed that the loss of *Pgrmc1* expression increased the pIRE1α-to-IRE1α ratio, suggesting that the loss of *Pgrmc1* expression could increase apoptosis by activating pIRE1α and deactivating IRE1α. When ATF4 in the PERK pathway is activated by eIF2α phosphorylation, it moves to the nucleus and induces the expression of genes encoding proteins involved in the unfolded protein response, including those related to ER stress-mediated apoptosis (49, 53). We confirmed that the liver of *Pgrmc1* KO mice fed the liquid EtOH diet showed high levels of phosphorylated eIF2α, increased ATF4 activity, and apoptosis. In addition, the ATF6 levels increased in the liver of *Pgrmc1* KO mice fed the liquid EtOH diet. We expected that the increase in ATF6 expression in *Pgrmc1* KO mice was associated with oxidative stress-induced apoptosis (13). As the increase in ATF6 expression increases cell death through the activation of GRP78 (48, 54, 55), the loss of *Pgrmc1* expression can be considered to induce susceptibility to alcohol-induced apoptosis. As a result, *Pgrmc1* KO mice are prone to apoptosis via the activation of cleaved caspase-3 and cleaved PARP.

The activation of the NF-κB pathway is associated with apoptosis-induced liver damage caused in ALD (56). NF-κB is activated by ACA (57) and regulates gene expression in various cellular processes, such as immune responses, cell proliferation, and apoptosis, and stress responses to toxic stimuli (58). The primary pathway activated by various inflammatory signals leads to the expression of IL-1β and TNF-α (59). The interaction between IRE1α, TRAF2, and IKK in ER stress plays an important role in NF-κB activation. It has been reported that IRE1α is involved in ER stress-induced inflammasome activation and IL-1β production, which play an important role in the transcription of inflammatory genes, because of its interaction with TRAF2 promoting NF-κB activation and inflammatory response (60, 61). Our results show that the pNF-κB-to-NF-κB ratio and *Il-1b* and *Tnf* expression were higher in *Pgrmc1* KO mice than in WT mice. In summary, the loss of *Pgrmc1* expression activates ER stress and increases susceptibility to the effects of alcohol, which induces cell death and may cause severe liver damage.

Under constant alcohol stress, cell components are damaged and antioxidant proteins are suppressed. As an important medium of cell defense, Nrf2 is activated and contributes to the cellular responses to oxidative stress and inflammation (62). In this study, *Pgrmc1* KO mice had lower *Nrf2* and *Sod1* mRNA levels than WT mice. Therefore, the loss of Pgrmc1 expression reduces the protection of liver cells against liver damage in vivo.

In summary, the loss of *Pgrmc1* expression increased ACA production by inducing the expression of alcohol-degrading enzymes, and the excess ACA consequently increased ER stress. Since high levels of ACA and ER stress in *Pgrmc1* KO hepatocytes promoted cell death and impaired homeostasis, the loss of *Pgrmc1* expression may increase susceptibility to ALD.

## DATA AVAILABILITY

Data will be made available upon reasonable request.

## SUPPLEMENTAL DATA

10.6084/m9.figshare.22262350.v1 Supplemental Figs. S1 and S2 and Supplemental Tables S1 and S2: https://doi.org/10.6084/m9.figshare.22262350.v1.

## GRANTS

This research was supported by the Basic Science Research Program through the National Research Foundation of Korea (NRF) funded by the Ministry of Education (2021R1I1A2042991). This work was also supported by an NRF grant funded by the Korean government (MSIT) (2021R1A4A1033078).

## DISCLOSURES

No conflicts of interest, financial or otherwise, are declared by the authors.

## AUTHOR CONTRIBUTIONS

S.L.J. and E.-J.H. conceived and designed research; S.L.J., I.-J.B., J.-W.K., and H.-J.S. performed experiments; S.L.J., J.-W.K., H.-J.K., H.-J.S., and E.-J.H. analyzed data; S.L.J. and I.-J.B. interpreted results of experiments; S.L.J., H.-J.K., H.-J.S., and E.-J.H. prepared figures; S.L.J. and E.-J.H. drafted manuscript; S.L.J., I.-J.B., J.-W.K., H.-J.K., H.-J.S., and E.-J.H. edited and revised manuscript; S.L.J., I.-J.B., J.-W.K., H.-J.K., and E.-J.H. approved final version of manuscript.
